# Antenatal Maternal Hemoglobin Level and Severe Maternal Morbidity

**DOI:** 10.3390/jcm14165823

**Published:** 2025-08-18

**Authors:** Sonya P. Fabricant, Karen N. Opara, Alesandra R. Rau, Julianna V. Paul, Alodia Girma, Jessica D. White, Gabriella Blissett, Intira Sriprasert, Lisa M. Korst, Nicole M. Chadwick

**Affiliations:** 1 Department of Obstetrics and Gynecology, Cedars-Sinai Medical Center, Los Angeles, CA 90048, USA; 2 Department of Obstetrics & Gynecology, The University of Texas Health Science Center, Houston, TX 77030, USA; 3 Irvine School of Medicine, University of California, Irvine, CA 92617, USA; 4 Los Angeles General Medical Center, University of Southern California, Los Angeles, CA 90033, USAintira.sriprasert@med.usc.edu (I.S.); nchadwick@dhs.lacounty.gov (N.M.C.); 5 San Diego School of Medicine, University of California, San Diego, CA 92093, USA; 6 Medical Campus, University of Colorado Anschutz, Aurora, CO 80045, USA; 7 Childbirth Research Associates, LLC, North Hollywood, CA 91607, USA

**Keywords:** anemia, severe maternal morbidity, maternal mortality, obstetrics, childbirth

## Abstract

**Background/Objectives**: Prior studies using administrative data have found that antenatal anemia is a risk factor for severe maternal morbidity (SMM). However, administrative definitions, including the commonly used definition from the Centers for Disease Control and Prevention (CDC), have a poor positive predictive value for some SMM components. We tested the relationship between hemoglobin level at delivery admission and SMM, as defined by gold-standard chart review. **Methods**: This was a retrospective case–control study of deliveries at a high-acuity hospital in Los Angeles, California, from 2016 to 2019. Administrative data were screened to identify patients with CDC SMM. Control-patients were selected at random from screen-negative individuals. Medical records for all individuals were reviewed for gold-standard SMM criteria, and clinical data were abstracted. Confirmed-positive and confirmed-negative patients were compared using bivariate analyses. Multiple logistic regression models were developed to test the relationship between admission hemoglobin level and gold-standard SMM. **Results**: Of 4202 eligible individuals, 275 (6.5%) screened positive for SMM. Of these, 107 (38.5%) met gold-standard SMM criteria; 285 confirmed-negative controls were retained for analysis. Case-patients were more likely than control-patients to have anemia on delivery admission (43.9% vs. 24.2%, *p* < 0.01) and had lower admission hemoglobin levels (11.2 ± 1.7 g/dL vs. 11.9 ± 1.3 g/dL, *p* < 0.01). After controlling for covariates, admission hemoglobin was independently and inversely associated with gold-standard SMM (aOR = 0.76, 95% CI 0.60–0.96, *p* = 0.02). **Conclusions**: Lower hemoglobin level at delivery admission was associated with an increased risk of developing gold-standard SMM.

## 1. Introduction

Severe maternal morbidity (SMM) is a composite measure of adverse outcomes that represent a “near-miss” for maternal mortality. Anemia has been identified as one potential risk factor for SMM [1,2,3,4,5]. The majority of prior studies examining this relationship have relied on administrative coding (such as the International Classification of Diseases, Tenth Revision, Clinical Modification [ICD-10], and Current Procedural Terminology [CPT] coding systems) used in medical billing and hospital discharge records to identify cases of SMM [1,2,3,4]. However, administrative definitions have a poor positive predictive value for some SMM components and risk overestimating the association between anemia and SMM [6,7]. This is particularly true for maternal morbidity related to hemorrhage and associated blood transfusion because administrative data do not document the number of units given. A commonly used SMM definition from the Centers for Disease Control and Prevention (CDC) is based on administrative data; using this definition, transfusion of as little as one unit of red blood cells (RBCs) meets SMM criteria [8]. In comparison, in-depth medical record review is considered the gold-standard for identifying cases of SMM [6,9].

Anemia is both highly prevalent in the obstetric population—affecting an estimated 11.5% of pregnancies in the United States [10]—and potentially modifiable. A better understanding of its relationship with maternal morbidity could inform strategies to improve maternal health outcomes. We performed a case–control study of deliveries at a high-acuity hospital in Los Angeles, California, to test the relationship between maternal hemoglobin at delivery admission and gold-standard SMM. We hypothesized that a lower admission hemoglobin level would be associated with increased risk of SMM.

## 2. Materials and Methods

This was a study of all childbirth admissions to Los Angeles County + University of Southern California Hospital from 2016 to 2019. Data for this cohort were provided by the California Maternal Quality Care Collaborative (CMQCC) Maternal Data Center [11] and consisted of linked patient-level hospital discharge and birth certificate data. Deliveries < 20 gestational weeks, pregnancy terminations, and deliveries with missing or erroneous information were excluded from the study cohort.

**Figure 1:**
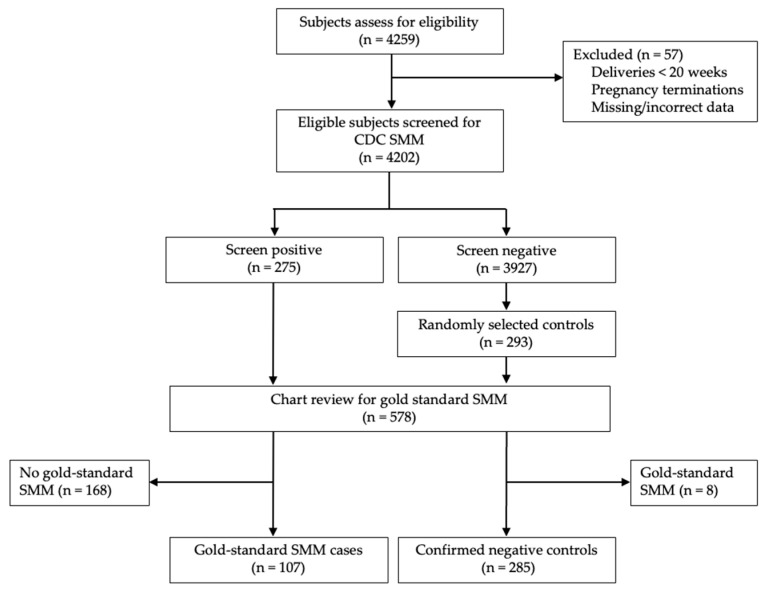
Flow diagram of eligible patients.

Eligible patients from the CMQCC cohort were screened for SMM using the CDC definition [8] (Figure 1). This definition includes 21 adverse outcomes, each defined by ICD-10 codes. Coding for CDC SMM was obtained courtesy of Fridman et al. [12]. All screen-positive patients were included as potential cases. A similar number of potential control-patients were randomly selected from screen-negative deliveries in the study period.

Medical records for screen-positive and screen-negative individuals were reviewed for the presence of clinical gold-standard criteria as defined by Main et al. [6]. This gold-standard definition is composed of 21 adverse outcomes previously validated by a combination of hospital quality review and expert consensus. A comparison of CDC SMM and gold-standard SMM definitions is provided in Appendix A. For example, using the CDC SMM definition, an individual meets criteria for SMM if they have an ICD-10 code for blood product transfusion during their delivery admission. In comparison, the gold-standard definition used in this study requires transfusion of 4 or more units of blood product, or any transfusion plus associated procedures/interventions (e.g., hysterectomy or uterine artery embolization) [6].

Data regarding antepartum hemoglobin levels were obtained from medical record review. Admission hemoglobin was defined as the hemoglobin level at the time of admission to the labor and delivery unit for childbirth. Presence of anemia was determined based on admission hemoglobin and defined as first- and third-trimester hemoglobin ≤ 11 g/dL, and second-trimester hemoglobin ≤ 10.5 g/dL [13]. Anemia was further subcategorized as mild (hemoglobin 9.0–10.5 g/dL in the second trimester and 9.0–11.0 g/dL in the third trimester), moderate (hemoglobin 7.0–8.9 g/dL), or severe (hemoglobin < 7.0 g/dL) [14]. SMM risk factors were previously described in a scoping review [15] and were identified for our patient population using administrative data. The remaining baseline patient characteristics were also obtained using administrative data, including patient age, race, ethnicity, body mass index (BMI), insurance type, and pregnancy history and characteristics.

Case-patients were screen-positive individuals with gold-standard SMM confirmed on chart review. Control-patients included only screen-negative individuals with gold-standard SMM excluded on chart review. Bivariate analyses were used to compare baseline characteristics and hemoglobin levels between cases and controls and to compare the frequency of individual gold-standard SMM components between cases with and without anemia.

Multiple logistic regression models were developed to evaluate the relationship between delivery admission hemoglobin level and gold-standard SMM. Backward, forward, and stepwise selection methods were used, with gold-standard SMM case status as the outcome and antepartum risk factors for SMM as covariates. Risk factors were included as covariates in the models based on the following criteria from bivariate analyses: *p* < 0.15, a maximum of 5 missing patients, and a minimum of 5 patients per predictor group. Only covariates that contributed to the model (*p* < 0.05) were retained. Black race was forced in the models as black race is known to increase risk for both SMM and anemia [16,17]. After evaluating the association with hemoglobin level, the models were re-run to test the associations between the outcome and (1) anemia (yes/no) and (2) anemia severity (none vs. mild vs. moderate/severe).

Seven investigators participated in the chart review. All underwent training to ensure abstraction consistency. Interrater reliability for 12 test records was found to be in very good agreement: kappa = 0.92 (95% CI 0.89 to 0.94). All calculations were performed using SAS (v 9.4, SAS Institute Inc., Cary, NC, USA). Categorical data were presented as number (percentage), and continuous data were presented as mean ± standard deviation (SD) or median (range). Odds ratios (ORs) and 95% confidence intervals (CIs) were reported. A *p*-value < 0.05 was considered statistically significant. This study was exempt by the Institutional Review Board at the University of Southern California Health Sciences Campus (Protocol #APP-19-06341).

## 3. Results

Of 4259 individuals who delivered in the study period, 4202 met the inclusion criteria. Screening with the CDC SMM definition identified 275 individuals (6.5%). Among these, 107 cases of gold-standard SMM (38.9%) were confirmed on chart review. Among 293 randomly selected controls, 8 (2.7%) were excluded for meeting gold-standard SMM criteria on chart review. The final analytic sample consisted of 107 cases and 285 controls.

Baseline characteristics were compared between cases and controls (Table 1). Case-patients were less likely to have a government payer (91.6% vs. 97.3%, *p* < 0.01). Groups were otherwise similar with regard to demographic characteristics. Regarding obstetric characteristics and comorbidities, case-patients were more likely to deliver preterm or by cesarean section. Case-patients were also more likely to have a multifetal gestation, fetal growth restriction, placenta previa or accreta spectrum disorder, pre-eclampsia, and medical comorbidities including cardiac, pulmonary, renal, and thyroid disease.

With regard to anemia characteristics, case-patients had lower mean admission hemoglobin levels (11.2 ± 1.7 g/dL vs. 11.9 ± 1.3 g/dL, *p* < 0.01) and were significantly more likely to have anemia on delivery admission (43.9% vs. 24.2%, *p* < 0.01) (Table 1). Case-patients were also more likely to have moderate or severe anemia compared to control-patients (moderate: 8.4% vs. 2.5%; severe: 0.9% vs. 0.0%; *p* < 0.01).

The frequency of SMM components was compared between case-patients with and without anemia (Table 2). The most common indicators for SMM among those with anemia included blood transfusion and pre-eclampsia. Those with anemia were more likely to receive a gold-standard blood transfusion (38.3% vs. 18.3%, *p* = 0.03) and were less likely to have gold-standard pre-eclampsia, though this finding did not achieve statistical significance (38.3 vs. 58.3%, *p* = 0.05). There was no other statistically significant difference between groups with regard to the frequency of SMM components. It is important to note that case numbers for individual SMM components were small, and the study was not powered to detect a difference in the individual components.

A multiple logistic regression model was developed to evaluate the independent effect of admission hemoglobin on case status. Admission hemoglobin was independently and inversely associated with SMM in the adjusted model (OR 0.76, 95% CI 0.60–0.96, *p* = 0.02) (Table 3). Other patient characteristics associated with SMM included preterm delivery, abnormal placentation, maternal cardiac disease, maternal renal disease, and gestational hypertension/pre-eclampsia. The c-statistic was 0.91. The Hosmer–Lemeshow test had a *p*-value of 0.74, indicating that the model was well-calibrated. Neither anemia as a categorical variable nor anemia severity contributed significantly to the model.

## 4. Discussion

In this case–control study, we demonstrated an inverse and independent relationship between delivery admission hemoglobin and gold-standard SMM. After controlling for relevant covariates, we found that for every one-point increase in hemoglobin level, a subject was 24% less likely to develop SMM.

Our findings are consistent with one prior study evaluating the relationship between anemia and gold-standard SMM: Guignard et al. identified SMM cases by medical record review using a definition derived from Delphi consensus. They found that anemia was associated with an increased odds of severe maternal morbidity among deliveries in France (aOR 1.8, 95% CI 1.5–2.1) [5].

The majority of other studies examining the relationship between anemia and SMM have used the CDC’s administrative definition of SMM [1,2,3,4], in which one unit of RBCs would qualify as SMM. However, transfusion of one unit of RBCs may not represent true maternal morbidity [9]. Indeed, a proactive approach to transfusion may represent efforts to reduce maternal morbidity. An alternative analytic approach is to test the relationship between administrative SMM and anemia but exclude the codes for blood transfusion [3]. Yet an assessment of the relationship between anemia and SMM would be incomplete without considering transfusion: blood transfusion represents a surrogate for hemorrhage, a leading cause of maternal morbidity and mortality [18]. We previously found that excluding transfusion codes from the CDC definition would miss 27.3% of gold-standard SMM cases [7]. Our use of a gold-standard definition of transfusion (large volume transfusion and/or need for procedural interventions such as hysterectomy) permitted identification of true clinical maternal morbidity related to hemorrhage.

Prior studies have found a greater risk of SMM with increasing anemia severity [2,5]. In our bivariate analysis, individuals with moderate or severe anemia were more likely to develop SMM. However, this association was no longer statistically significant after adjustment for confounders. It is important to note that our study was not powered to detect differences in SMM risk by anemia severity.

Comparing rates of SMM components between case-patients with and without anemia, we found that pre-eclampsia accounted for a greater proportion of SMM cases among those without anemia, though this did not achieve statistical significance (*p* = 0.05). We suspect our results are attributable to the timing of hemoglobin level ascertainment in our population. We evaluated hemoglobin level on delivery admission, a time when patients are likely to be presenting in the acute stages of pre-eclampsia. Acute pre-eclampsia can lead to intravascular depletion and hemoconcentration, with a subsequent spurious increase in hemoglobin [19]. Besides pre-eclampsia and transfusion, there was no difference in the rate of individual SMM components between cases with and without anemia, though our study was not powered to detect a difference for the individual components.

The relationship between anemia and SMM is likely multifactorial and extends beyond an increased risk for blood transfusion. Lower red blood cell volume compromises hemostatic mechanisms and the ability to compensate for bleeding during delivery, increasing the risk of postpartum hemorrhage, coagulopathy, and hysterectomy [1,5,20,21]. Anemia has also been identified as an independent risk factor for infection (including sepsis) [1,20,22], cesarean delivery [20], and hypertensive disorders of pregnancy [1,4,20,23]. Finally, anemia may be a surrogate for other factors that predispose to SMM, including inflammation, infectious disease, malnutrition, and socioeconomic deprivation/barriers to care [21,22,24].

Predicting individuals at risk for SMM poses an ongoing challenge to providers [25]. Based on our findings, anemia at delivery admission may serve as an early warning sign for increased risk of rare but serious maternal complications. Recognizing this association can help providers prioritize prompt clinical assessment of anemic patients who exhibit a clinical status change and facilitate the early mobilization of the multidisciplinary obstetric team if needed.

Targeted interventions to prevent SMM in individuals with anemia should focus on the specific SMM components most relevant to this population. We found that gold-standard blood transfusion was among the most common indicators for SMM for individuals with anemia. Several interventions to address the risk of life-threatening hemorrhage in this group can be considered: First, hospital systems can adopt hemorrhage risk assessment tools that incorporate anemia into the risk calculation and ensure these tools are standardized across delivery admissions. Second, providers can ensure adequate preparation for hemorrhage, including ready access to uterotonics, tranexamic acid, and intrauterine compression devices. Finally, providers can ensure immediate blood product availability in the event that a life-threatening hemorrhage does occur. The CMQCC Obstetric Hemorrhage Safety Bundle is one potential tool that uses hemoglobin level at childbirth admission to guide hemorrhage risk stratification, prevention, and management [26]. This tool has been shown to reduce rates of SMM overall and mitigate racial disparities in SMM [27].

Prenatal interventions to identify and treat anemia are another important consideration when discussing tools to prevent SMM. Society guidelines recommend anemia testing and treatment beginning at the first prenatal visit based on the physiological demands of pregnancy and the observed association between anemia and individual adverse pregnancy outcomes [28]. However, there remains insufficient direct evidence to determine the impact of anemia treatment on composite SMM [28], and more research is needed to guide recommendations in this area.

Our study has a number of strengths. To our knowledge, ours is the first study to test the relationship between anemia and gold-standard SMM within a US cohort. Use of a gold-standard definition of SMM excluded false-positive SMM cases, thus preventing the risk of overestimating the association between SMM and anemia that can be seen with administrative definitions. Additionally, the study site was a county hospital primarily serving an underinsured and socioeconomically disadvantaged population, which may mitigate socioeconomic disadvantage as a confounder for the relationship between anemia and SMM.

Our study has several limitations. Using administrative data to screen for gold-standard SMM missed some SMM cases. Additionally, even though we limited the outcome and independent variable to data abstracted from the medical record, the remaining SMM risk factors were obtained from administrative data, and thus, the limits of using these data apply to our analyses [29]. Finally, admission hemoglobin was not categorized to distinguish between those patients with antenatal anemia vs. those with an acute episode of bleeding prior to admission; the latter would also be more likely to experience SMM and could falsely elevate the estimated OR between admission hemoglobin and SMM.

## 5. Conclusions

Our study demonstrated that hemoglobin level on delivery admission was inversely related to the odds of developing gold-standard SMM. Anemia should be considered a red flag warning for patients at risk for developing adverse peripartum outcomes, including SMM. Providers should consider implementing standardized hemorrhage risk assessment and prevention tools to help reduce the risk of hemorrhage-related SMM in this population. Research is needed to investigate whether antenatal anemia treatment can reduce rates of composite SMM.

## Figures and Tables

**Table 1:** Baseline and hemoglobin characteristics of individuals with and without gold-standard severe maternal morbidity.

Baseline Characteristics	Cases (N = 107)	Controls (N = 285)	*p* *^α^*
Age, y			
<20	8 (7.5%)	27 (9.5%)	0.81
20–34	75 (70.1%)	198 (69.5%)	
≥35	24 (22.4%)	60 (21.1%)	
Race			
Asian/Pacific Islander	4 (3.7%)	15 (5.3%)	0.84
Black (non-Hispanic)	14 (13.1%)	30 (10.5%)	
White (non-Hispanic)	42 (39.3%)	115 (40.4%)	
Hispanic	44 (41.1%)	118 (41.4%)	
Other	1 (0.9%)	1 (0.4%)	
Unknown	2 (1.9%)	6 (2.1%)	
Gestational age at delivery			
≥37 weeks	39 (36.4%)	231 (81.2%)	<0.01
32 to <37 weeks	41 (38.3%)	43 (15.1%)	
<32 weeks	27 (25.2%)	11 (3.9%)	
Pre-pregnancy body mass index (kg/m^2^)	29.4 ± 9.1 (n = 101)	28.1 ± 6.7 (n = 275)	0.49
Public health insurance	98 (91.6%)	284 (97.3%)	**<0.01**
Nulliparous	44 (41.9%) (n = 105)	114 (40.3%) (n = 283)	0.82
Cesarean delivery	86 (80.4%)	98 (34.4%)	**<0.0001**
Fetal growth restriction	18 (16.8%)	19 (6.7%)	**<0.01**
Prior cesarean delivery	41 (38.3%)	72 (25.3%)	**0.01**
Multifetal gestation	7 (6.5%)	2 (0.7%)	**<0.01**
Fetal malpresentation	25 (23.4%)	22 (7.7%)	**<0.01**
Placenta previa or accreta spectrum	12 (11.2%)	4 (1.4%)	**<0.01**
Cardiac disease	28 (26.2%)	6 (2.1%)	**<0.01**
Pulmonary disease	20 (18.7%)	23 (8.1%)	**<0.01**
Renal disease	20 (18.7%)	5 (1.8%)	**<0.01**
Thyroid disease	14 (13.1%)	13 (4.6%)	**<0.01**
Bowel disease	12 (11.2%)	16 (5.6%)	0.08
Gestational diabetes	12 (11.2%)	22 (7.7%)	0.31
Pre-gestational diabetes	12 (11.2%)	15 (5.3%)	0.05
Gestational hypertension or pre-eclampsia	63 (58.9%)	47 (16.5%)	**<0.01**
Group B streptococcus colonization	13 (12.1%)	54 (18.9%)	0.13
Human immunodeficiency virus infection	13 (12.1%)	20 (7.0%)	0.11
Hemoglobin level on delivery admission	11.2 ± 1.7 (95% CI: 10.9–11.5)	11.9 ± 1.3 (95% CI: 11.7–12.0)	**<0.01**
Anemia on delivery admission ^ϕ^	47 (43.9%)	69 (24.2%)	**<0.01**
None	60 (56.1%)	216 (75.8%)	**<0.01**
Mild	37 (34.6%)	62 (21.8%)	
Moderate	9 (8.4%)	7 (2.5%)	
Severe	1 (0.9%)	0 (0%)	

Data presented as number (percentage) or mean ± standard deviation. CI, confidence interval. ***^α^*** Bold values indicate statistical significance (*p* < 0.05). ^ϕ^ Anemia: 1st- and 3rd-trimester hgb < 11 g/dL, 2nd-trimester hgb < 10.5 g/d; mild: 2nd-trimester hgb 9.0–10.5 g/dL or 3rd-trimester hgb 9.0–11.0 g/dL, moderate: hgb 7.0–8.9 g/dL, severe: hgb < 7.0 g/dL.

**Table 2:** Gold-standard severe maternal morbidity in individuals with and without anemia.

Severe Maternal Morbidity Component	Anemia (N = 47)	No Anemia (N = 60)	*p* *^α^*
Acute myocardial infarction	0	0	-
Aneurysm	1 (2.1%)	2 (3.3%)	1.00
Renal failure	4 (8.5%)	4 (6.7%)	0.73
ARDS	6 (12.8%)	4 (6.7%)	0.33
Amniotic fluid embolism	0	0	-
Cardiac arrest	0	0	-
Conversion cardiac rhythm	1 (2.1%)	0	0.44
DIC	2 (4.3%)	1 (1.7%)	0.58
Pre-eclampsia	18 (38.3%)	35 (58.3%)	0.05
Cardiovascular complications	1 (2.1%)	3 (5.0%)	0.63
Pulmonary edema/acute heart failure	4 (8.5%)	6 (10.0%)	1.00
Anesthesia complications	1 (2.1%)	0 (0%)	0.44
Sepsis	3 (6.4%)	5 (8.3%)	1.00
Shock	3 (6.4%)	4 (6.7%)	1.00
Sickle cell crisis	1 (2.1%)	0 (0%)	0.44
Venous thromboembolism	0 (0%)	1 (1.7%)	1.00
Hysterectomy	3 (6.4%)	2 (3.3%)	0.65
Mechanical ventilation	3 (6.4%)	1 (1.7%)	0.32
Blood transfusion (gold standard)	18 (38.3%)	11 (18.3%)	**0.03**

Data are presented as number (percentage). ***^α^*** Bold values indicate statistical significance (*p* < 0.05). ARDS, acute respiratory distress syndrome. DIC, disseminated intravascular coagulopathy.

**Table 3:** Multiple logistic regression model for severe maternal morbidity by admission hemoglobin.

Variable	Unadjusted OR (95% CI)	Adjusted OR (95% CI)	*p* *^α^*
Admission hemoglobin	0.71 (0.61–0.84)	0.76 (0.01–0.95)	**0.02**
Black race	1.28 (0.65–2.52)	1.78 (0.74–4.28)	**0.20**
Preterm delivery (<37 weeks)			
Preterm delivery (32 0/7–36 6/7 weeks)	5.81 (3.36–10.04)	4.47 (2.17–9.19)	**<0.0001**
Very preterm delivery (<32 weeks)	17.18 (7.49–39.43)	13.4 (5.16–34.8)	**<0.0001**
Placenta previa or accreta spectrum	8.87 (2.80–28.16)	8.84 (2.36–33.0)	**<0.01**
Cardiac disease	16.5 (6.6–41.2)	38.0 (12.0–119.8)	**<0.0001**
Renal disease	12.9 (4.7–35.3)	16.5 (4.61–58.8)	**<0.0001**
Gestational hypertension/pre-eclampsia	7.3 (4.4–11.9)	6.13 (3.20–11.7)	**<0.0001**

c-statistic = 0.91. ***^α^*** Bold values indicate statistical significance (*p* < 0.05). OR, odds ratio; CI, confidence interval.

## Data Availability

Data is available upon request to the corresponding author.

## Abbreviations

The following abbreviations are used in this manuscript:

CDC	Centers for Disease Control and Prevention
CI	Confidence interval
CMQCC	California Maternal Quality Care Collaborative
ICD10	International Classification of Diseases, Tenth Revision, Clinical Modification
OR	Odds ratio
RBC	Red blood cell
SMM	Severe maternal morbidity

## Appendix A

**Table A1:** Indicators for severe maternal morbidity (SMM).

CDC SMM Indicators [8]	Gold-Standard SMM Indicators from Main et al. [6]
Acute myocardial infarction	Acute myocardial infarction
Aneurysm	Aneurysm
Acute renal failure	Acute renal failure Acute Tubular Necrosis Dialysis Oliguria treated with multiple doses of Lasix Creatinine > 2.0 without pre-existing renal disease Doubling of baseline creatinine with pre-existing renal disease
Adult respiratory distress syndrome	Adult respiratory distress syndrome ARDS, pulmonary edema, or postoperative pneumonia Requiring ventilation
Amniotic fluid embolism	Amniotic fluid embolism
Cardiac arrest/ventricular fibrillation	Cardiac arrest/ventricular fibrillation
Conversion of cardiac rhythm	Conversion of cardiac rhythm Arrhythmia that requires > 1 dose of IV medication but not ICU admission Arrhythmia that requires ICU admission with further treatments Central line or pulmonary catheter placement to monitor for a complication
Disseminated intravascular coagulation	Disseminated intravascular coagulation
Eclampsia	Eclampsia and severe pre-eclampsia Pre-eclampsia with difficult-to-control severe hypertension with blood pressure >160/110 that requires multiple IV doses (more than 1) or IV drip, or remains persistent >48 h after delivery Liver or subcapsular hematoma Severe liver injury admitted to ICU (bilirubin > 6, liver enzymes > 600) HELLP syndrome (severe hemolysis or coagulation abnormalities, elevated liver enzymes, and low platelet count) Non-responsiveness or loss of vision (temporary or permanent)
Heart failure/arrest during surgery or procedure	Heart failure/arrest during surgery or procedure Pre-existing cardiac disease with ICU admission for treatment Peripartum cardiomyopathy
Puerperal cerebrovascular disorders	Puerperal cerebrovascular disorders
Pulmonary edema/acute heart failure	Pulmonary edema/acute heart failure
Severe anesthesia complications	Severe anesthesia complications Total spinal anesthesia Aspiration pneumonia Epidural hematoma
Sepsis	Sepsis Hypotension with multiple liters of IV fluids or pressors Pulmonary edema or ARDS
Shock	Shock
Sickle cell disease with crisis	Sickle cell disease with crisis
Air and thrombotic embolism	Air and thrombotic embolism
Blood product transfusion	Blood product transfusion Obstetric hemorrhage with ≥4 units of red blood cells transfused Obstetric hemorrhage with 2 units of red blood cells and 2 units of fresh frozen plasma transfused (without other procedures or complications), if not judged to be “over-exuberant” transfusion Obstetric hemorrhage with <4 units of blood products transfused and evidence of pulmonary congestion that requires >1 dose of Lasix Obstetric hemorrhage with return to the operating room for any major procedure, excluding dilation and curettage Any emergency/unplanned peripartum hysterectomy, regardless of number of units transfused Obstetric hemorrhage with uterine artery embolization regardless of number of units transfused Obstetric hemorrhage with uterine balloon or uterine compression suture placed and 2–3 units of blood products transfused Obstetric hemorrhage admitted to the intensive care unit for invasive monitoring or treatment
Hysterectomy	Hysterectomy and surgical complications Bowel injury (beyond minor serosal tear) Bladder injury (beyond minor serosal tear) Small Bowel obstruction during pregnancy/postpartum period Prolonged ileus > 4 days
Temporary tracheostomy	Temporary tracheostomy
Ventilation	Ventilation (other than ventilation for a surgical procedure)

ARDS, acute respiratory distress syndrome; IV, intravenous; ICU, intensive care unit; HELLP, hemolysis, elevated liver enzymes, and low platelets.
